# A Comprehensive Literature Review on the Effects of Formaldehyde on the Upper Respiratory Tract

**DOI:** 10.7759/cureus.59743

**Published:** 2024-05-06

**Authors:** Harriet Kaye Austin, Erik Schoenberg

**Affiliations:** 1 Otolaryngology, University of Central Florida College of Medicine, Orlando, USA; 2 Otolaryngology, HCA Florida Orlando Allergy and ENT, Sanford, USA

**Keywords:** mucociliary clearance, respiratory epithelium, histopathological findings, indoor air pollution, upper respiratory tract infection, upper respiratory tract, formaldehyde exposure

## Abstract

Prolonged exposure to indoor air pollutants at high concentrations can have adverse health effects on the respiratory system of individuals who spend most of their time indoors. Formaldehyde (FA) is a common indoor air pollutant because of its extensive use in household products such as cleaners, floorings, and furnishings. As a chemical, FA is highly water soluble and reactive. When its airborne form is inhaled, it is mainly absorbed in the upper airways. FA has been extensively studied for its carcinogenic effects, but it can also cause inflammation in the upper airways. The objective of the current review was to assess the secondary effects of such inflammation and how it can contribute to an increased risk for upper respiratory infections, which are mostly caused by viruses. A rigorous literature review was conducted through gathering, reading, and analyzing relevant literature, including peer-reviewed articles published after 1990 and seminal literature regardless of publication date. Findings from the review provide a greater understanding of the outcomes of FA exposure, the potential accumulative damage to the upper respiratory tract, and the associated increased risk for acute infections of the upper respiratory tract. This information can help in the development and enforcement of stricter regulations for furniture and building materials for household-related products to limit exposure to indoor pollutants such as FA.

## Introduction and background

Indoor air pollutants and their effects on the upper respiratory tract (URT)

Exposure through inhalation of air pollutants, including indoor pollutants such as formaldehyde (FA), can lead to inflammation of the respiratory system [1-3]. Studies have shown that inflammation plays a major role in the development of numerous chronic diseases and disorders [2]. In addition, the imbalance between antioxidants in the body and free radicals from various environmental exposures can lead to the progression of inflammation-associated illnesses, including greater susceptibility to infections [4,5]. In particular, the upregulation of pro-inflammatory molecules such as cytokines as an inflammatory response may be associated with the dysfunction of respiratory cilia and mucus retention [6-8]. Consequently, the inflammatory response triggered by air pollutants may be a factor in the increased risk of upper respiratory tract infections (URIs). 

URIs are defined by irritation and swelling of the upper airways, specifically, the nose, pharynx, and larynx, that are unrelated to pre-existing respiratory conditions [9]. The infections are commonly caused by viruses and bacteria that invade the mucosal structures of the upper airways. For microorganisms to invade and infect, they must be able to adhere to the cell walls before colonizing. The respiratory tract has several barriers and defense mechanisms that serve as protection against the outside environment. The mucus secretions in the respiratory tract usually trap pathogens/microorganisms and prevent them from continuing further into the system [9]. In addition, the cilia lining the respiratory tract also aid in trapping organisms. Further, the mucociliary escalator transports harmful microorganisms back up to the pharynx, which also serves as a clearance process. 

The mucociliary structures in the respiratory tract also serve as a protective barrier against gases. Mucus flow plays a role in the removal of inhaled FA, so any changes to it could affect the metabolism of FA. FA can react with macromolecules such as proteins and nucleic acids, and it has been found to be associated with inflammatory processes and oxidative stress [5,10]. FA is classified as an indoor air pollutant because ambient air concentrations of FA are much lower (1-4 μg/m^3^), with the exception of major cities. Indoor environments have numerous sources of FA, including wood products, furniture, foam insulation, textiles, paints, varnishes, electronic equipment, and even cosmetic products [11,12]. Due to the ubiquitous presence of FA in household products, the majority of individuals are continuously exposed to some amount of it, and there are many ways that FA can contribute to indoor concentrations if not regulated and monitored [13]. Indoor FA emissions are dependent on the wood products and other indoor sources, temperature, humidity, and the ventilation of the building [14]. 

FA and its role as an indoor air pollutant

In previous animal studies, only a small percentage of FA was shown to reach the lower airways and trigger airway inflammation [15]. Since inhalation is the major route of exposure, almost 95% of inhaled FA is retained in the upper airways; therefore, its effects are limited to the site of contact, which will mainly be the upper airways [16]. This limitation is supported by studies that showed no significant changes in lung function associated with FA exposure [17-19]. The lung function tests, also known as pulmonary function tests, used in these studies included forced vital capacity (FVC) and forced expiratory volume in the first second (FEV1) [20]; the ratio of FEV1/FVC is typically used to differentiate between various types of lung diseases. Values for the lung function tests are used to determine whether any abnormalities in the respiratory function are present and to quantify the severity of pulmonary disease [21]. However, the pulmonary function tests alone are not used for specific diagnosis [20,21]. 

Moreover, physical characteristics, such as the size, of particulate matter contribute to its mechanism of deposition and toxicity. Particles that are greater than 5 μm tend to be limited to deposition in the upper and larger airways [22]. To affect the smaller airways and alveoli, particles must be 2-5 μm or less than 2 μm, respectively [22]. FA that tends to be released from wood particles that are greater than 6 μm will mostly affect the URT as it will be unable to reach the smaller, lower airways.

On average, FA concentrations are less than 0.05 mg/m^3^ in homes; however, newer buildings and furnishings are associated with concentrations that can exceed 0.2 mg/m^3^ [23]. Higher temperatures, increased humidity, tobacco smoke, and ozone-initiated reactions can also contribute to higher levels of FA. However, particle formation is negligible if the FA concentration is kept at or below 0.4 mg/m^3^ [11]. 

Human studies completed in China that examined the effects of long-term occupational exposure to FA found changes in the nasal mucosa, including but not limited to inflammation, hyperplasia, chronic rhinitis, and pharyngitis, along with other sensory irritation [24-26]. Prior to 1990, studies that examined cellular changes from short-term and long-term exposure to inhaled FA found increased cellular proliferation in the nasal mucosa at concentrations of 2 ppm or higher [27,28]. Increased cellular proliferation indicates that the nasal epithelium has been damaged and repair is underway to restore the mucosa to its normal state. Animal studies examining the effects of chronic exposure showed inflammation of the mucosa, epithelial dysplasia, and metaplasia [29,30]. 

Limitations in assessing the effects of FA

Establishing a definitive relationship between FA and its effect on the upper airways is difficult due to confounding variables, such as co-pollutants that contribute to inflammation and irritation of the upper airways. FA is classified by the International Agency for Research on Cancer (IARC) as a Group 1 human carcinogen, and there is sufficient evidence to link FA exposure with nasopharyngeal cancer and myeloid leukemia [31]. A Group 1 IARC classification indicates sufficient evidence that FA is carcinogenic in experimental animals and strong evidence that it is involved in the mechanism of carcinogenicity in exposed humans. 

Based on human and animal studies, ocular and URT irritation begins at any FA exposure greater than 0.1 ppm [32]. However, irritation is not the best toxic endpoint for determining the exposure limits. Human studies were completed to determine exposure concentrations using sensory irritation as its primary criteria, but the data were inconclusive and could not be used to estimate the risk of prolonged exposure to FA. Observing cellular changes in animal studies in conjunction with human studies on sensory irritation could be used as an alternative approach when assessing the risks of non-cancer-related effects, such as the effects on the upper airways. 

An expert panel suggested that irritation can be prevented in all individuals, including those who are more sensitive to irritants, if the indoor air concentrations of FA are kept below 0.1 ppm [33]. However, it is important to establish strict indoor guidelines for FA because its main route of exposure is through indoor air pollution and high concentrations can possibly lead to damaging health effects. In addition to indoor guidelines, other ways to control concentrations of FA are to promote the use of low-emission products, especially household-related products, and to improve indoor ventilation. 

**Figure 1:**
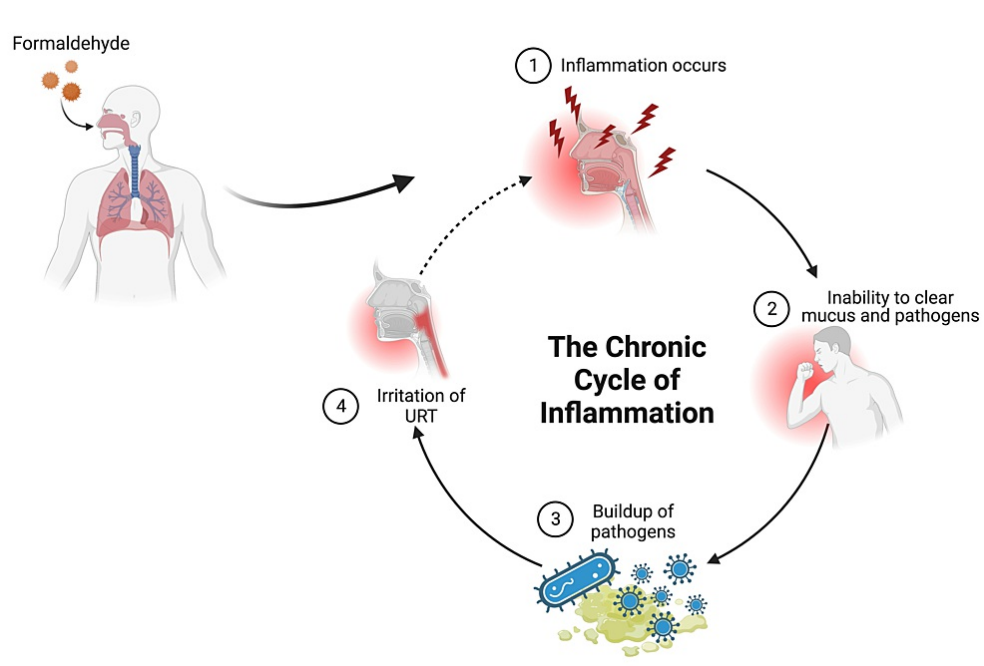
Chronic cycle of inflammation secondary to formaldehyde. Created by authors using BioRender. URT: upper respiratory tract

Along with studies that compare the effects of prolonged exposure to low FA concentrations and acute exposures to high concentrations, more research is needed to determine whether FA causes histopathological changes in the URT. Such changes are indicative of nasal and upper airway injury, which could cause people to become more susceptible to infections, particularly URIs, as shown in the pathway depicted in Figure 1. The research objective is thus to determine how FA can disturb the integrity of the upper respiratory mucosa without using cancer and sensory irritation as an endpoint.

Methods

A literature search was conducted to identify relevant studies, and their results and methodologies were summarized and compared to provide a better understanding of how FA exposure alters nasal function and inflammatory responses. Based on previous studies, assessments of nasal cytology, nasal lavage (NAL), ciliary and clearance function through the ciliary beat frequency (CBF), and mucociliary clearance (MC) are optimal for assessing such changes. These changes can potentially be associated with the hypothesized increased risk of infections, owing to the weakening of the respiratory tract defense mechanisms against pathogens [34,35]. The amount of FA exposure was reviewed from in vitro and in vivo experimental studies, while measured environmental FA exposures were reviewed from human observational studies [36-46].

A computer-assisted exhaustive literature search, primarily of Google Scholar and PubMed databases, was conducted using the snowballing method (forward and backward). The snowballing method consists of consulting the bibliography of key articles to find other relevant publications. The following keywords were used in several combinations to begin the search process: (1) URT, (2) NAL, (3) ciliary functions, (4) nasal epithelium, (5) FA, (6) indoor air pollution, (7) inflammatory markers, and (8) inflammation. Existing studies and other publications that fit the inclusion criteria were then examined to determine the excess risk of developing respiratory infections due to higher exposure to FA relative to negligible FA exposure. 

The following inclusion criteria were used to identify publications for the literature review: (1) studies relating to FA exposure, (2) peer-reviewed articles published after 1990 with the exception of seminal or landmark literature, (3) studies must be original, (4) studies must use objective measures, (4) animal studies were performed using an experimental study design, (5) human studies were conducted in a self-controlled case series, (6) human studies were conducted with a population age of 18 years or more, and (7) human studies could include men and women. 

Publications meeting the following exclusion criteria were not included in the literature review: (1) reviews and editorials, (2) data on individuals with chronic conditions, (3) data on individuals with a history of comorbidities, (4) gray literature, (5) data based on self-reported measures, such as studies that used qualitative methodology, and (6) study populations located in heavily polluted areas. This last criterion was applied to minimize confounding effects due to traffic exhaust and other common air pollutants, such as particulate matter.

## Review

**Figure 2:**
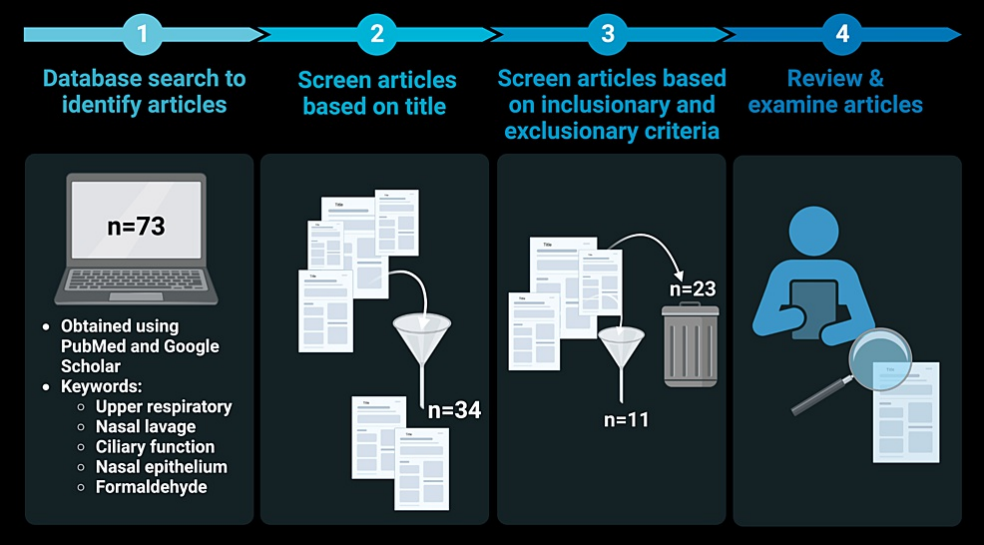
Methodology diagram of literature review study. Created by authors using BioRender.

Seventy-three articles were initially found using PubMed and Google Scholar using the keywords noted in the Methods section. Subsequently, the articles were filtered based on the title, and 34 of those articles were deemed to be the most relevant. After screening the articles based on the inclusion and exclusion criteria, only 11 studies were included in the literature review (Figure 2). FA is well studied regarding its toxicology and mechanism of action as a carcinogen, but epidemiological studies to quantify its association with URIs are lacking. During the literature review, there was only one epidemiological study on the relationship between FA exposure and respiratory infections; however, the focus in that study was lower respiratory tract infections in infants [47]. As the results did not relate to the URT, the study was not included in the literature review; however, it is notable that the results indicated an increase of lower respiratory tract infections in infants that were exposed to FA. This finding would be especially concerning for parents moving into new homes that may have higher FA emissions. 

**Figure 3:**
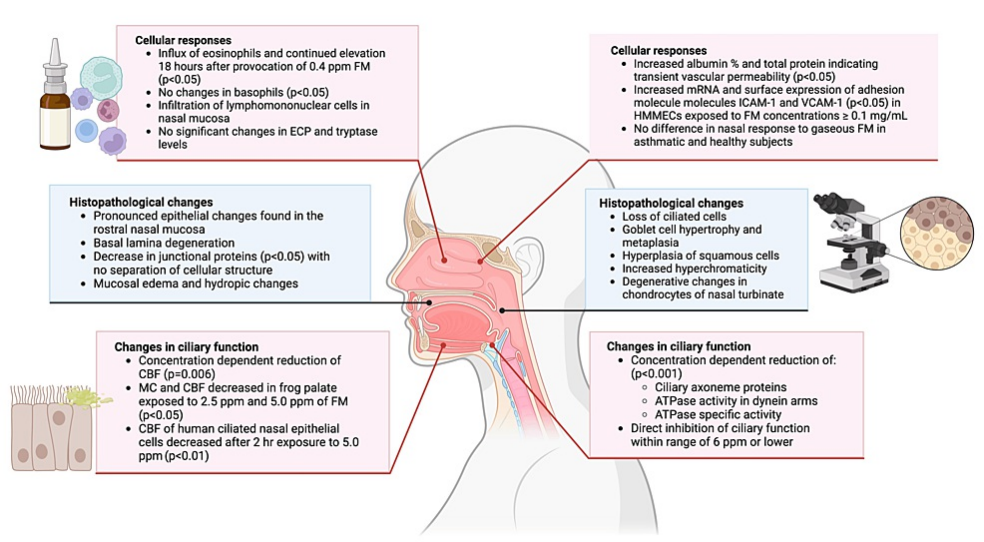
Graphic that summarizes significant findings of the studies. Created by authors using BioRender. FM: formaldehyde; MC: mucociliary clearance; CBF: ciliary beat frequency; HMMECs: human mucosal microvascular endothelial cells; ECP: eosinophil cationic protein

Due to its reactive nature, gaseous FA is typically metabolized and absorbed by the mucosal linings of the URT and rarely goes further than the major bronchi if the concentrations are low enough. However, concern remains regarding whether FA exposure has an impact on the incidence of URIs. The selected articles reviewed in Table 1 and summarized in Figure 3 provide significant results, indicating that FA can disrupt the defense mechanisms of the URT and thus potentially lead to increased susceptibility to microorganisms and URIs. 

The MC is an important protective process that depends on functioning cilia and proper mucus production. Disruption of either component can lead to impairment of the harmonic movement that clears out pathogens. Studies that examined CBF in the trachea were included in the literature review, as the MC includes both the collaboration of the upper and lower respiratory tract cilia and mucus production to function properly. Damage to the cilia motility functions in the lower respiratory tract, in this case, the trachea, could indirectly affect how the MC can remove pathogens from the respiratory tract in general, allowing microorganisms to accumulate and cause infections. Tracheal changes included atrophic changes in the epithelial lining, with cellular disorganization, ulcerations, squamous metaplastic changes (likely as a response to the irritation from FA), and ulcerations; increased goblet cells; and deciliation and clumping of cilia, which would impair the functional activity of cilia required for the continuous movement of secretions towards the pharynx. Based on cellular responses, FA results in nonspecific nonallergic inflammatory processes in the nasal mucosa. Notable evidence for these processes is the lack of eosinophilic cationic protein and high tryptase levels that are usually seen in mast cell and eosinophil degeneration [48]. Other notable findings included a decrease of spontaneous neutrophil burst activity observed in occupationally exposed workers with URT findings and a history of recurrent URIs. These observations were potentially due to FA exposure and increased individual susceptibility owing to dysfunction in the ability to regenerate microbicidal reactive oxygen species that would normally kill invading microorganisms. Moreover, supporting this finding is the lack of differences found in nasal response to gaseous FA in asthmatic and healthy subjects. Many other studies confirmed the occurrence of epithelial changes, such as hyperplasia and squamous metaplasia of the nasal respiratory epithelium [49]. 

Studies that examined systemic effects of FA exposure beyond the URT were not included in the review, but it is worth mentioning findings that support increased inflammation as a result of FA exposure. For example, Oztan et al. [50] reported a positive correlation in levels of the pro-inflammatory cytokines TNF-a and IL-6 with increasing exposures of FA based on blood samples from occupationally exposed workers (p<0.001). However, it is unclear how this could directly affect the URT [50]. 

Discussion and limitations

The majority of the recent literature I encountered during the course of my literature review focused on the short-term effects of high levels of FA rather than the cumulative effects of chronic low exposure to FA. The latter is more applicable to FA in the real world as an indoor air pollutant. Although FA exposure was controlled, 54% of the selected studies were conducted in vitro (39% in animals and 15% in humans), while 46% were conducted in vivo (23% in animals and 23% in humans). Consequently, there is difficulty in establishing the exact concentration of FA in the airway fluid at any given inhaled concentration due to variable factors related to the conditions during tidal breathing, such as the frequency and pattern of respiration (nasal versus mouth). Such variation can distort the true effects of FA on the URT with respect to the concentration of exposure. Furthermore, it is important to consider differences in the effects of gaseous FA and FA attached to particles.

**Table 1:** Descriptive summary of studies included in the literature review. FA: formaldehyde; H&E: hematoxylin and eosin; HMMECs: human mucosal microvascular endothelial cells; PAS: periodic acid-Schiff; RT-PCR: reverse transcription-polymerase chain reaction; SDS-PAGE: sodium dodecyl-sulfate polyacrylamide gel electrophoresis *: p<0.05; **: p<0.01

Reference	Methods	Type of exposure	FA concentrations	Subject	Measured variables
Hastie et al. [36]	High-speed video microscopy	Solubilized FA	0, 37.5, 75, and 150 mg/mL	Rabbit tracheal explants in vitro	Ciliary beat frequency (Hz)
	BioRad protein microassay, Fiske and SubbaRow method, SDS-PAGE			Porcine tracheal explants in vitro	Ciliary axonemes: protein concentration, ATPase activity, and protein profile
Fló-Neyret et al. [37]	Stereoscopic microscope	Solubilized FA	1.25, 2.5,* and 5.0* ppm	Isolated frog palate in vitro	Mucociliary clearance
	High-speed video microscopy				Ciliary beat frequency
Colizzo et al. [38]	BioRad protein microassay, Fiske and SubbaRow method, and SDS-PAGE	Solubilized FA	0, 37.5, 75, and 150 mg/mL	Bovine tracheae in vitro	Ciliary axonemes: protein concentration, ATPase activity, and protein profile
	Light microscopy and high-speed video microscopy			Rabbit trachea in vitro	Ciliary activity
Arican et al. [39]	Light microscopy (H&E and PAS stain)	Gaseous FA	15 ppm	Rats in vivo	Histopathological changes in the nasal epithelium
	Electron microscopy				
	Immunohistochemistry				Junctional protein changes of the nasal respiratory mucosa
Bansal et al. [40]	Light microscopy (H&E stain)	Formalin (solubilized FA)	10% and 40% FA solutions	Rabbits in vivo	Histopathological changes in the nasal cavity and trachea and lungs
	Electron microscopy				
Bruno et al. [41]	Nasal cytology	Occupational exposure	Mean levels ranged from <0.04 to 0.15 ppm	FA-exposed workers	Alterations in the nasal mucosa
Krakowiak et al. [42]	Nasal lavage	Gaseous FA	0.5 mg/m^3^	Human in vivo	FA-induced nasal responses
Pazdrak et al. [43]	Nasal lavage	Gaseous FA	0.5 mg/m^3^	Human in vivo	FA-induced nasal responses
Kim et al. [44]	Flow cytometry RT-PCR	Gaseous FA	0.001, 0.01, 0.1,* and 1* mg/m^3^	HMMECs from nasal mucosal cells	Changes in the expression of adhesion molecules (ICAM-1 and VCAM-1)
	Eosinophil adhesion assay		0.001, 0.01, 0.1,** and 1** mg/m^3^		Adhesiveness between HMMECs and eosinophils
Speit et al. [45]	Light microscopy (H&E stain)	Gaseous FA	0, 0.5,* 1,** 2,* 6,** 10,** and 15** ppm	Rats in vivo	Histopathological changes in the nasal cavity
	Immunostaining with anti-BrdUrd-antibody				Cellular proliferation
Schäfer et al. [46]	High-speed video microscopy	Gaseous FA	100, 500, and 5,000** mg/m^3^	Human ciliated nasal epithelial cells in vitro	Ciliary beat frequency

The components essential in MC are properly functioning cilia and appropriate mucus production. Notably, one study that assessed the effects of FA on frog mucociliary epithelium created three groups of specimens exposed to progressively increased concentrations of FA of 1.25 ppm, 2.50 ppm, and 5.0 ppm, respectively [37]. Groups 2 and 3 were shown to have decreased MC and significantly decreased CBF compared to group 1 within 60 minutes of exposure. Group 1 had increased CBF in comparison to other groups including the control, but MC was impaired due to inharmonious movements of cilia. The observed CBF is likely the cilia trying to compensate for impaired harmonic movements of cilia. On the other hand, group 2 had higher toxic effects as shown by the simultaneous decrease in both CBF and MC leading to mucociliary function impairment. 

Of note, there is no evidence of mast cell and eosinophil degranulation which indicates nonspecific nonallergic inflammatory processes in the nasal mucosa. In the same study, it shows no significant changes in eosinophil cationic protein and tryptase levels which are typically indicators of allergic reaction [42]. While these results indicate that FA may not be involved in the process of allergic inflammation, it is still unclear whether it plays a role in sensitizing the respiratory system. 

According to the Environmental Protection Agency (EPA), homes with significant amounts of newly pressed wood products can have FA levels greater than 0.3 ppm. However, warmer temperatures and high humidity levels can further increase FA emissions. Although the World Health Organization has set health-based indoor air quality guidelines for FA (along with other indoor air pollutants) and the EPA regulates FA emission standards in composite wood products, there are no ventilation guidelines/standards to manage the concentration of FA indoors [51]. Even at low doses of 0.5 mg/m^3^, data indicate that FA has irritative effects and can promote nonspecific pro-inflammatory properties [42,43]. Investing in the improvement of ventilation systems in new buildings could potentially mitigate the economic burden related to poor health outcomes linked to indoor air pollutants such as FA. 

This literature review suggests the need for epidemiological studies to determine whether a relationship exists between FA exposure and increased incidence of URIs. Due to the known carcinogenicity nature of FA, it would be difficult and unethical to perform an experimental study on the effects of isolated exposure to FA in a controlled setting. Epidemiology studies would be the best alternative to quantify the association between FA exposure and acute upper respiratory infections. However, this would present with a problem of FA being confounded with other air pollutants which would make it difficult to assess whether FA alone is associated with increased respiratory infections. 

The limitations of this study encompass several factors. Firstly, there is a notable language bias, as only studies published in English were included, potentially excluding relevant research published in other languages. Additionally, the scope and depth of the review were constrained by the limited number of articles that met the predefined inclusion criteria, totaling only 11 articles. This restricted sample size may have hindered the comprehensive exploration of the topic. Furthermore, the generalizability of the findings may be limited due to the predominant focus on in vitro and in vivo animal studies within the included literature. These studies may not fully capture the complexities and variations present in human subjects or clinical settings. Lastly, the reliance on only two databases for literature search increases the possibility of overlooking relevant studies that may exist in other databases or sources. Despite these limitations, this study provides valuable insights within its defined parameters, but caution should be exercised when interpreting and applying the findings to broader contexts. This study's key strength lies in its capacity to directly inform policy-making through evidence-based insights and recommendations derived from a rigorous literature review. These insights empower policymakers to make informed decisions regarding stricter regulations on FA in household products or enhancing ventilation systems, develop targeted interventions, and allocate resources effectively to address societal issues. Additionally, by identifying research gaps and methodological limitations, the study guides future research endeavors, contributing to ongoing knowledge advancement. Overall, its practical application bridges the gap between research and policy, facilitating effective decision-making processes for policymakers.

## Conclusions

FA, a well-known carcinogen and prevalent indoor air pollutant found in household products, poses risks of chronic inhalation leading to URT injuries. These injuries may increase susceptibility to infections, with acute respiratory infections being a significant outpatient and inpatient concern. Understanding indoor air quality as a risk factor for such injuries is crucial. However, due to limited studies on chronic low-level exposure, the cumulative effects remain unclear. This literature review focuses on histopathological and immunological changes associated with FA exposure, particularly highlighting its impact on mucociliary function, a vital defense mechanism against upper respiratory infections. It underscores the importance of recognizing FA's toxic effects and its potential to induce inflammation upon exposure. Future perspectives should address the need for more comprehensive studies to better understand the long-term effects of FA exposure on human health.
